# Legislating novel psychoactive substances: lessons from 15 years of UK mortality data (2007-2022)

**DOI:** 10.3389/fphar.2025.1708335

**Published:** 2026-01-29

**Authors:** Kirsten L. Rock, Ric Treble, Caroline S. Copeland

**Affiliations:** 1 Institute of Pharmaceutical Sciences, King’s College London, London, United Kingdom; 2 Retired, London, United Kingdom; 3 National Programme on Substance Use Mortality, London, United Kingdom

**Keywords:** novel psychoactive substances, drug policy, international drug control, opioids, stimulants, cannabinoids

## Abstract

**Background:**

Novel Psychoactive Substances (NPS) emerged in the early 2000s as chemically designed alternatives to circumvent laws which internationally control drugs. There is limited evidence that NPS are produced in the UK, whereas China has long been recognised as a primary source of NPS. This study aimed to evaluate the relative impact of UK, Chinese, and UN legislative controls on the availability of NPS in the UK, as evidenced by post-mortem detections of NPS in deaths.

**Methods:**

Deaths reported to the National Programme on Substance Use Mortality (NPSUM) which occurred 2007–2022 were extracted for analysis. Drugs from the three major substance classes–opioids, stimulants and cannabinoids–which were detected in these deaths were categorised according to their control status as either classical substances (i.e., those under international control prior to 2007), NPS controlled in China, or other NPS.

**Findings:**

Across all three drug classes, detections of classical substances dominated throughout the study period. Detections of NPS opioids–primarily fentanyl analogues–peaked in 2017, NPS stimulants–notably cathinones–in 2015, and synthetic cannabinoid receptor agonists in 2018 and 2021. Whilst UK legislative controls (the Misuse of Drugs Act 1971, Temporary Class Drug Orders, the Psychoactive Substances Act 2016) were generally implemented first, reductions in NPS detections were more closely associated with the introduction of Chinese legislations - in particular the 2021 Chinese generic ban on synthetic cannabinoids which resulted in an almost complete disappearance of these compounds in UK deaths in 2022.

**Conclusion:**

The findings of this study indicate that the most effective way to reduce NPS availability in the UK is via legislation in producer countries, as evidenced by substantial declines in their detections in deaths following their control in China. This reliance on international controls places the UK in a vulnerable position, as its domestic drug landscape is being shaped largely by the pace and scope of independent international legislations. To achieve and maximise effectiveness, UK drug policy needs to integrate harm reduction measures alongside the introduction of legislative controls, whilst also encouraging international efforts to bring in global control of problem materials.

## Highlights

- Producer-country controls appear to be more effective than domestic legislation.
- UK demand-side drug policy interventions appear to have had limited impact.
- Control of NPS may be driving substitution to more harmful substances.

## Introduction

Novel psychoactive substances (NPS) are drugs that have been newly designed to mimic the effects of traditional controlled substances (e.g., opioid, stimulants) (Deen et al., 2021). They pose significant health risks due to their unknown potencies, toxic effects, and lack of pre-clinical and clinical testing (Norman and Lee, 2017). There is very little evidence that NPS are manufactured within the United Kingdom (UK), with the UK NPS market instead supplied by imports of materials synthesised overseas: China has long been recognised as a primary source of NPS (Zhao, 2022; Zhao, 2020; Bao et al., 2019; Deventer Marie et al., 2025; Seddon, 2014; Gomes and Rudkowsky, 2025), with the products of its extensive and proficient chemical industry infrastructure now readily available to the rest of the world by means of internet trading and rapid postal delivery services (Zhao, 2022; Seddon, 2014; Gomes and Rudkowsky, 2025).

### Legal controls on NPS

Controls on NPS have been progressively introduced as new materials have emerged and evidence of their harms has accumulated (Deen et al., 2021). Individual countries are responsible for legislative control of drugs within their jurisdiction. Additionally, there is overarching international drug control co-ordinated by the United Nations (UN), intended to provide a globally consistent approach (United Nations Office on Drugs and Crime, 1961; United Nations Office, 1971; United Nations Office on Drugs and Crime, 1988). UN guidance on materials for control are required to be enacted into national legislations of member states (United Nations Office on Drugs and Crime, 1961; United Nations Office, 1971; United Nations Office on Drugs and Crime, 1988).

### UN guidance on regulation of NPS

The UN operates three major international Conventions addressing drug control: the 1961 Convention on Narcotic Drugs; the 1971 Convention on Psychotropic Substances; the 1988 Convention against Illicit Traffic in Narcotic Drugs and Psychotropic Substances (United Nations Office on Drugs and Crime, 1961; United Nations Office, 1971; United Nations Office on Drugs and Crime, 1988). These are updated annually by the UN Commission on Narcotic Drugs (CND), based on recommendations from the World Health Organisation’s (WHO) Expert Committee on Drug Dependence (ECDD). Since 2015, the annual extensions of the Conventions’ lists have primarily concerned NPS and their precursors.

### UK legislative control of NPS

The UK as a UN member state is obliged to place substances listed in the three international drug Conventions under national drug control. The UK has also controlled many additional psychoactive substances which are considered to represent a hazard to human health (UK Government, 1971; UK Government, 2016).

UK drug controls are primarily enacted by the Misuse of Drugs Act 1971 (the MDA (UK Government, 1971)). In addition to specific substances, the MDA also includes generic controls on families of psychoactive substances with a common core chemical structure intended to control ‘designer drugs’ – variants of controlled drugs chemically ‘designed’ so as to avoid specifically worded legislative controls (UK Government, 1971).

From 2009, as NPS began to emerge, a series of substance-specific and generic controls were brought into effect by means of additions to the MDA (e.g., synthetic cannabinoid receptor agonists [SCRAs], cathinones) (UK Government, 1971). However, such MDA additions took time to implement as evidence of substance-related harms needed to be gathered. The MDA was therefore supplemented by the use of emergency Temporary Class Drug Orders (TCDOs), introduced in November 2011 to permit more rapid control of NPS whilst their potential harms were more fully evaluated (Home Office, 2011).

However, as both the MDA and TCDOs require the identification of specific substances (or families of substances) to bring them under control, this proved a significant limitation to the control of rapidly emerging NPS. In response, the Psychoactive Substances Act 2016 (the PSA) was brought into effect in late May 2016 (UK Government, 2016). The PSA includes provisions to prevent the importation, distribution, and sale of any psychoactive substances which were outside the scope of the MDA and TCDO controls, unless specifically exempted (UK Government, 2016). This had the immediate effect of outlawing the open sale of NPS through commercial outlets such as ‘head shops’ and internet vendors (Home Office, 2018).

### Chinese legislative control of NPS

China is also a UN member state and therefore a signatory of the three major international drug Conventions (United Nations Office on Drugs and Crime, 1961; United Nations Office, 1971; United Nations Office on Drugs and Crime, 1988). Similarly to the UK, China has also enacted a series of additional controls on a large number of specified NPS (Ministry of Public Security of the People’s Republic of China, 2015; Ministry of Public Security of the People’s Republic of China, 2018) as well as a broad generic ban on fentanyl derivatives in May 2019 (Ministry of Public Security of the People’s Republic of China, 2019) and a set of generic controls on many SCRA structural families in May 2021 (Ministry of Public Security of the People’s Republic of China, 2021).

### Evolution of NPS

As substance-specific legislative controls have been introduced, NPS producers have rapidly adjusted their products or developed new NPS based on core structures not yet addressed by international or national controls (Deen et al., 2021; Groth et al., 2022; Halter et al., 2020; Jalal and Burke, 2021). This iterative process of new NPS, substance-specific legal controls, and rapid replacement by further NPS has become a game of “cat and mouse” or “whack-a-mole” (Gomes and Rudkowsky, 2025; Hall and Miczek, 2019).

### Study aim and method of investigation

In this study we aimed to evaluate the relative impact of UK, Chinese and UN control measures on NPS available on the UK illicit drug market. This evaluation was based on the most serious adverse effect of NPS, as evidenced by post-mortem detections of NPS in deaths from England, Wales and Northern Ireland recorded by the National Programme on Substance Use Mortality (NPSUM) over a 15-year period, from 2007 to 2022.

## Methods

### National programme on substance use mortality (NPSUM)

The NPSUM receives voluntary reports from over 85% of English, Welsh, Northern Irish and Islands’ (Jersey, Guernsey, Isle of Man) coroners on deaths related to psychoactive drugs. A death is referred to a coroner if it has an unknown cause, is violent or unnatural, sudden, and unexplained, occurred during an operation or before the person came out of an anaesthetic, or potentially caused by an industrial disease or poisoning (gov.uk, 2025). Toxicology tests are requested dependent upon individual case circumstances at the discretion of the coroner and consulting pathologist.

A range of documents comprise coronial inquest files, although this varies from case to case. Typically, the coroner has access to: statements from witnesses, family and friends; General Practitioner (GP) records (if the deceased is registered with one); reports from first responders (e.g., police, emergency services); hospital emergency departments and clinical ward reports; psychiatric and substance abuse team reports; as well as post-mortem and toxicology reports. Information from these reports is transposed into the relevant data fields on the NPSUM database as either numerical variables or string text, as appropriate. All drugs detected by toxicological testing of post-mortem samples are entered on the NPSAD database, except for caffeine and nicotine (and metabolites thereof).

The King’s College London Biomedical and Health Sciences, Dentistry, Medicine and Natural and Mathematical Sciences Research Ethics Subcommittee re-confirmed in August 2025 that NPSUM does not require research ethics committee review as all subjects are deceased.

### Case identification

A retrospective study design was used to identify all cases with detections of opioids, stimulants, or cannabinoids in post-mortem tissue (e.g., blood, urine, vitreous humor, stomach contents, liver, skeletal muscle) which occurred in the 15-year period 2007–2022 and were reported to the NPSUM by 1 November 2024. These three drug classes were chosen as the majority of additional controls enacted by the UK, China and UN have focused upon substances from within these classes (United Nations Office on Drugs and Crime, 1961; UK Government, 1971; Ministry of Public Security of the People’s Republic of China, 2015; Ministry of Public Security of the People’s Republic of China, 2018; Ministry of Public Security of the People’s Republic of China, 2019; Ministry of Public Security of the People’s Republic of China, 2021). Sub-analysis was then performed on cases with detections of opioids, stimulants, or cannabinoids which had been controlled in China between 2015 and 2021 either directly via legislation introduced by the Council of China, or indirectly via their addition to the International Drug Control Conventions by the UN Commission on Narcotic Drugs.

In the vast majority of cases, NPS detections were made from preserved blood and urine samples. UK toxicology laboratories typically screen for drugs using an immunoassay or a multi-analyte liquid chromatography–mass spectrometry (LC–MS) screening technique (Rooney et al., 2023). The samples then proceed to confirmatory testing using either an LC–MS/gas chromatography–mass spectrometry (GC–MS) or high-resolution accurate mass (HRAM) analysis (Rooney et al., 2023).

### Data analysis

*Software:* Data analysis were performed using IBM® SPSS™ Statistics for Windows version 31, with visualisations constructed using Microsoft Excel 365.

*Statistics:* Given the time-series nature of the data and the close temporal proximity of when the UK, Chinese and UN NPS legislations were introduced, formal statistical testing was deemed to not be appropriate. The analyses are therefore descriptive, focusing on the temporal relationship between NPS detections in deaths and when the various NPS legislations were introduced.

## Results

This study has been subdivided into three broad categories: opioids, stimulants, and cannabinoids. In the tables (Tables 1–3), the substances listed within each category have been segmented according to whether or not they were considered ‘classical’ substances. Classical substances are those that were under international control prior to the start of the study period (i.e., 2007). All other substances within each drug class have then been further segmented as to if and when they became controlled by China, and coloured coded as to when each was controlled by the UK, China and UN either in response to international guidance being issued or by means of a country-specific additional control.

**TABLE 1:** Detections of opioids in deaths reported to the NPSUM 2007–2022 subdivided by type (classical opioids; NPS opioids controlled in China in 2015, 2017, 2019, 2021; other opioids) and colour coded with year and jurisdiction of control according to the Colour Wheel of Drug Policy (yellow: UN; blue: UK; red: China; orange: UN and China; green: UN and UK; purple: UK and China; grey: UN, UK and China).

Drug	Year																
	2007	2008	2009	2010	2011	2012	2013	2014	2015	2016	2017	2018	2019	2020	2021	2022	
Classical opioids																	
Alfentanil	0	0	1	0	1	1	1	2	0	2	4	2	3	4	7	13	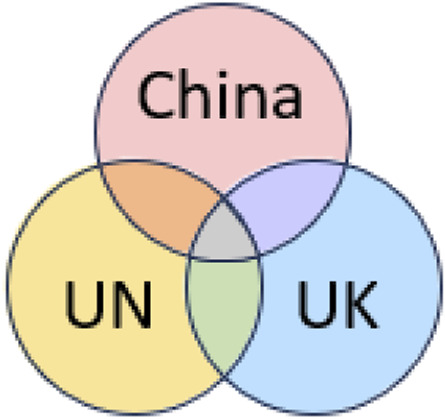
Buprenorphine	8	6	11	15	12	12	27	36	41	34	52	63	82	104	101	98	
Codeine	214	227	321	270	257	258	284	279	282	307	286	387	354	367	420	386	
Dextropropoxyphene^a^	42	21	20	10	5	4	8	5	3	1	2	1	1	0	3	1	
Dihydrocodeine	131	140	136	103	128	101	103	105	129	112	100	130	146	157	146	134	
Dihydromorphine	0	0	0	0	0	0	0	0	0	0	0	3	0	1	4	0	
Dipipanone	8	5	9	2	4	2	1	3	0	0	1	1	1	1	0	0	
Etonitazene	0	0	0	0	0	0	0	0	0	0	0	0	0	0	4	5	
Fentanyl	7	5	13	11	18	18	31	26	44	52	86	63	72	59	51	53	
Hydrocodone	1	2	4	2	1	5	5	1	4	3	5	15	24	51	48	33	
Levorphanol	0	0	0	0	0	0	1	1	1	0	0	2	0	0	1	0	
Meptazinol	2	4	0	1	0	1	0	0	1	0	1	5	0	2	3	3	
Methadone	380	369	437	390	446	345	353	331	398	382	401	478	577	856	826	793	
Morphine/Heroin	1029	1089	1305	812	672	677	674	844	990	991	931	1117	1380	1303	1295	1298	
Oxycodone	19	17	24	26	31	36	47	38	62	51	66	74	74	80	83	106	
Oxymorphone	0	0	0	0	0	0	0	0	4	2	1	1	4	7	2	0	
Pentazocine	0	0	1	0	0	0	0	0	0	0	0	0	0	0	0	0	
Pethidine	6	2	8	1	3	0	1	2	3	1	0	0	2	0	0	1	
Remifentanil	0	0	0	0	0	0	0	0	0	0	0	2	0	1	1	1	
Tramadol	94	83	125	130	182	157	208	194	198	183	173	199	224	240	205	223	
NPS opioids controlled in China in 2015																	
4-Fluorobutyrylfentanyl	0	0	0	0	0	0	0	0	1	0	6	2	0	0	0	0	
Acetylfentanyl	0	0	0	0	0	0	0	0	2	0	5	0	0	1	0	0	
AH-7921	0	0	0	0	0	0	3	1	0	0	0	0	0	0	0	0	
Butyrfentanyl	0	0	0	0	0	0	0	0	0	0	6	1	1	0	0	0	
Ocfentanil	0	0	0	0	0	0	0	0	0	0	1	0	1	0	0	0	
NPS opioids controlled in China in 2017																	
Carfentanil	0	0	0	0	0	0	0	0	0	1	51	0	0	0	0	0	
Furanylfentanyl	0	0	0	0	0	0	0	0	0	0	6	1	0	0	0	0	
U-47,700	0	0	0	0	0	0	0	0	0	1	0	0	0	0	0	0	
NPS opioids controlled in China in 2019																	
2-Fluorofentanyl	0	0	0	0	0	0	0	0	0	2	0	0	0	0	0	0	
Cyclopropylfentanyl	0	0	0	0	0	0	0	0	0	0	3	2	0	0	0	0	
Despropionyl fentanyl	0	0	0	0	0	0	0	0	0	0	3	3	1	1	0	0	
Fluorofentanyl (unspecified)	0	0	0	0	0	0	0	0	0	1	0	1	0	0	0	0	
Methoxyacetylfentanyl	0	0	0	0	0	0	0	0	0	0	1	7	0	2	1	0	
NPS opioids controlled in China in 2021																	
Isotonitazene	0	0	0	0	0	0	0	0	0	0	0	0	1	0	17	2	
Other opioids																	
Brorphine	0	0	0	0	0	0	0	0	0	0	0	0	0	0	1	0	
Etodesnitazene	0	0	0	0	0	0	0	0	0	0	0	0	0	0	1	2	
Kratom/Mitragynine	0	0	0	0	0	4	2	0	4	1	0	0	1	0	1	1	
N-Pyrollidino etonitazene	0	0	0	0	0	0	0	0	0	0	0	0	0	0	4	8	
Protonitazene	0	0	0	0	0	0	0	0	0	0	0	0	0	0	0	1	
Tapentadol	0	0	0	0	0	0	2	2	1	2	5	8	15	13	10	20	
Unidentified synthetic opioid	0	0	0	0	0	0	0	0	0	0	0	1	0	0	0	0	

^a^ Considered as a licensed medication as previously licensed and prescribed.

**TABLE 2:** Detections of stimulants in deaths reported to the NPSUM 2007–2022 subdivided by type (classical stimulants; NPS stimulants controlled in China in 2015, 2016, 2018, 2021; other stimulants) and colour coded with year and jurisdiction of control according to the Colour Wheel of Drug Policy (yellow: UN; blue: UK; red: China; orange: UN and China; green: UN and UK; purple: UK and China; grey: UN, UK and China).

Drug	Year																
	2007	2008	2009	2010	2011	2012	2013	2014	2015	2016	2017	2018	2019	2020	2021	2022	
Classical stimulants																	
Amphetamine	105	99	88	68	104	85	120	106	140	133	137	140	178	203	196	164	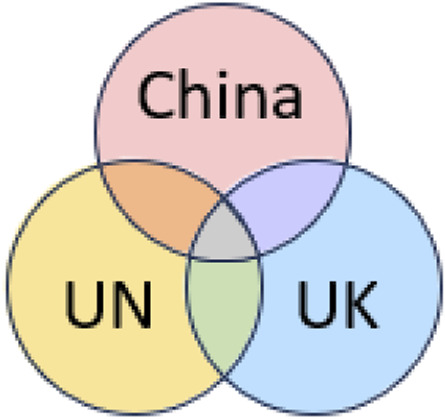
Cathine	0	3	1	0	0	1	0	1	1	1	1	2	1	0	7	1	
Cathinone	0	4	1	0	1	1	0	0	1	1	0	0	1	1	2	1	
Cocaine	427	422	341	232	236	264	335	398	502	597	756	938	1228	1371	1379	1779	
DMT	0	0	0	0	0	0	0	0	0	0	0	0	0	0	0	1	
Fenproporex	0	1	0	0	0	0	0	0	0	0	0	0	0	0	0	0	
Methamphetamine	6	6	3	3	6	7	10	11	8	11	15	17	25	37	43	37	
Methylphenidate	0	1	0	1	0	0	1	0	5	2	3	6	0	7	8	8	
MDMA	71	40	8	13	21	43	45	40	59	64	73	79	98	99	42	31	
MDA	0	1	0	0	0	1	1	0	0	0	1	0	0	0	1	1	
MDEA	0	0	0	0	1	0	0	0	1	1	0	0	1	5	2	2	
N-Ethylamphetamine	0	0	0	0	0	1	0	0	0	0	0	0	0	0	1	1	
Para-methoxyamphetamine	0	0	0	1	4	24	33	4	1	0	0	0	0	2	0	0	
Phentermine	1	0	0	0	0	1	0	1	0	0	0	0	0	0	1	0	
Pyrovalerone	0	0	0	1	0	0	0	0	0	0	0	0	0	1	0	0	
NPS stimulants controlled in China in 2015																	
2-AI	0	0	0	0	0	0	0	1	0	1	0	1	1	1	1	1	
2-Methoxyphenidine	0	0	0	0	0	0	0	6	12	5	0	0	0	0	0	0	
3-MMC	0	0	0	0	0	0	0	0	0	0	0	1	0	1	0	0	
3-Trifluoromethylphenylpiperazine	4	3	22	14	5	3	3	0	0	0	0	0	0	1	1	0	
4-Fluoroamphetamine	0	0	1	0	0	1	0	0	2	1	0	0	1	0	0	0	
4-Fluoromethamphetamine	0	0	0	0	0	0	0	0	0	0	0	0	1	0	0	0	
5-MAPB	0	0	0	0	0	0	1	1	0	0	0	0	0	0	0	0	
5-MeO-DALT	0	0	0	1	0	0	1	1	0	0	0	0	0	0	0	0	
5-MeO-DiPT	0	0	0	0	0	0	1	0	0	0	0	0	0	0	0	0	
Alpha-PHP	0	0	0	0	0	0	0	0	0	0	1	1	1	6	16	4	
Alpha-PVP	0	0	0	0	0	0	0	2	4	8	0	1	1	0	3	3	
AMT	0	0	0	0	2	3	5	5	3	0	0	0	0	0	0	0	
Benzylpiperazine	9	17	25	13	5	8	3	0	0	0	0	0	0	1	0	0	
Butylone	0	0	0	0	0	0	1	0	3	0	3	0	1	0	0	0	
Clephedrone	0	0	0	0	0	0	0	0	2	2	0	0	0	0	0	0	
Desoxypipradrol	0	0	0	3	0	0	0	0	0	0	0	0	0	0	0	0	
DOC	0	0	0	0	1	0	0	0	0	0	1	0	0	0	0	0	
Ethylone	0	0	0	0	0	0	1	2	3	0	0	0	0	1	0	0	
Ethylphenidate	0	0	0	0	0	0	3	20	10	1	0	1	0	0	1	0	
Flephedrone	0	0	0	2	2	3	0	0	2	0	0	0	0	0	0	0	
Fluoromethcathinone	0	0	0	0	0	0	2	2	1	0	0	0	0	0	0	0	
MCPP	0	0	0	0	0	0	1	0	0	0	1	1	1	2	3	3	
MDAI	0	0	0	0	2	1	0	0	0	0	0	0	0	0	0	0	
MDPBP	0	0	0	0	1	0	0	0	0	0	0	0	0	0	0	0	
MDPV	0	0	0	9	4	1	1	1	1	0	0	0	0	0	1	0	
Mephedrone	0	0	7	46	25	25	30	32	34	7	4	5	4	3	8	2	
Methedrone	0	0	0	2	3	0	0	0	0	0	0	0	0	0	1	2	
Methiopropamine	0	0	0	0	0	4	8	16	18	6	1	0	0	0	1	0	
Methylone	0	0	0	2	0	4	9	3	1	0	1	0	0	2	0	0	
Pentedrone	0	0	0	0	0	1	0	0	0	0	0	0	0	0	0	0	
NPS stimulants controlled in China in 2016																	
4-Methylethcathinone	0	0	0	0	5	14	13	6	5	2	1	3	0	0	0	0	
PMMA	0	0	0	0	1	3	6	0	0	0	0	0	0	1	0	0	
NPS stimulants controlled in China in 2018																	
4-Chloroethcathinone	0	0	0	0	0	0	0	0	0	0	2	2	0	0	0	0	
4C-PVP	0	0	0	0	0	0	0	0	0	0	1	0	0	0	0	0	
4-MEAPP	0	0	0	0	0	0	0	0	0	0	3	0	0	0	0	0	
Dibutylone	0	0	0	0	0	0	0	0	0	0	1	0	2	5	0	0	
Ephylone	0	0	0	0	0	0	0	0	0	1	4	2	1	0	0	0	
MDPHP	0	0	0	0	0	0	0	0	0	0	11	15	1	3	4	7	
Mexedrone	0	0	0	0	0	0	0	0	2	7	1	0	0	0	1	0	
Naphyrone	0	0	0	1	0	0	0	1	0	0	0	0	0	0	0	0	
N-Ethylhexedrone	0	0	0	0	0	0	0	0	0	0	1	0	0	0	0	0	
Pentylone	0	0	0	0	1	0	0	0	0	0	0	0	0	1	0	0	
NPS stimulants controlled in China in 2021																	
1,2-Diphenidine	0	0	0	0	0	0	0	2	1	1	0	0	0	0	0	0	
3-FPM	0	0	0	0	0	0	0	0	6	2	1	0	1	0	1	0	
3-MeO-PCP	0	0	0	0	0	0	0	0	0	0	0	0	0	1	0	0	
Eutylone	0	0	0	0	0	0	0	0	0	0	0	0	0	4	0	0	
Other stimulants																	
2-C-I	0	0	0	0	0	0	1	0	0	0	0	0	0	0	0	0	
3-FMC	0	0	0	0	1	0	0	0	0	0	0	0	0	0	0	0	
4-Ethylphenethylamine	0	0	0	0	0	0	1	0	0	0	0	0	0	0	0	1	
4-Fluoromethylphenidate	0	0	0	0	0	0	0	0	0	0	0	0	1	0	0	0	
4-Methylamphetamine	0	0	0	0	1	2	2	5	0	0	0	0	0	0	0	0	
5/6-APB	0	0	0	0	1	4	3	1	1	1	0	0	0	0	0	0	
5-IAI	0	0	0	0	0	1	0	1	0	1	0	0	0	0	0	0	
Aminoindane unspecified	0	0	0	0	0	0	0	0	1	1	0	0	0	0	0	0	
Atomoxetine^a^	0	0	0	0	0	0	0	0	0	1	0	0	0	0	0	0	
Benzedrone	0	0	0	0	0	0	0	0	0	0	0	0	0	0	0	1	
CPP	1	0	0	0	1	0	1	0	0	0	0	2	1	5	4	7	
Desoxy-D2PM	0	0	0	0	0	0	1	0	0	0	0	0	0	0	0	0	
DOI	0	0	0	0	0	0	1	0	0	0	0	0	0	0	0	0	
EAPB	0	0	0	0	0	0	0	1	0	1	0	0	0	0	0	0	
Fluorocathinone	0	0	0	0	0	0	0	0	1	0	0	0	0	0	0	0	
Fluoropiperazine	0	0	1	0	0	0	0	0	0	0	0	0	0	0	0	0	
Ibogaine	0	0	1	0	0	0	0	0	0	0	0	0	0	0	0	0	
Methcathinone unspecified	0	0	0	1	1	0	0	0	0	0	0	0	0	0	0	0	
Methoxypiperamide	0	0	0	0	0	0	0	0	1	1	0	0	0	0	0	0	
Modafinil^a^	0	0	1	0	0	0	0	1	0	1	3	2	4	1	4	3	
Phenylpiracetam^b^	0	0	0	0	0	0	0	0	0	0	1	0	0	0	0	0	
Tryptamine unspecified	0	0	1	0	0	0	0	0	0	0	0	0	0	1	1	0	

^a^ Psychoactive medications not subject to the PSA.

^b^ Shown to act on CNS receptors but not formally tested for psychoactivity at time of writing so cannot be considered banned under the PSA

**TABLE 3:** Detections of cannabinoids in deaths reported to the NPSUM 2007–2022 subdivided by type (cannabis [THC]; NPS synthetic cannabinoids controlled in China in 2015, 2018, 2021; other synthetic cannabinoids) and colour coded with year and jurisdiction of control according to the Colour Wheel of Drug Policy (yellow: UN; blue: UK; red: China; orange: UN and China; green: UN and UK; purple: UK and China; grey: UN, UK and China).

Drug	Year											
	2012	2013	2014	2015	2016	2017	2018	2019	2020	2021	2022	
Cannabis (THC)	148	201	171	216	241	289	370	463	518	511	512	
NPS synthetic cannabinoids controlled in China in 2015												
5F-AMB	0	0	0	0	0	0	0	3	1	1	0	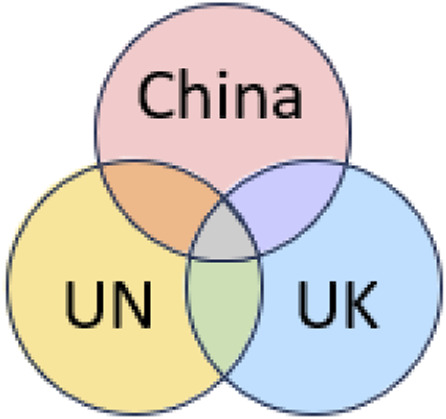
5F-APINACA	0	2	3	5	2	0	0	0	0	0	0	
5F-PB-22	0	1	3	3	1	0	1	2	1	0	0	
AB-CHMINACA	0	0	1	2	0	0	0	0	0	0	0	
AB-FUBINACA	0	0	1	0	3	14	19	3	3	1	0	
AB-PINACA	0	0	0	0	1	0	0	0	0	5	0	
AKB-48	0	0	1	0	0	0	0	0	0	0	0	
AM-2201	1	0	0	0	0	0	0	0	0	0	0	
AM-2233	1	0	0	0	0	0	0	0	0	0	0	
MDMB-CHMICA	0	0	0	5	2	4	0	0	0	0	0	
STS-135	0	0	3	0	0	0	0	0	0	0	0	
NPS synthetic cannabinoids controlled in China in 2018												
5F-ADB	0	0	0	0	8	37	50	5	3	1	1	
ADB/AMB/EMB-FUBINACA	0	0	0	0	0	4	4	0	0	3	0	
NPS synthetic cannabinoids controlled in China in 2021												
4F-ABUTINACA	0	0	0	0	0	0	0	0	2	9	0	
4F-AKB-48	0	0	0	0	0	0	0	0	0	1	0	
4F-MDMB-BICA	0	0	0	0	0	0	0	0	6	22	2	
4F-MDMB-BINACA	0	0	0	0	0	0	3	16	18	2	0	
5F-EMB-PICA	0	0	0	0	0	0	0	0	0	2	0	
5F-MDMB-PICA	0	0	0	0	0	0	1	22	14	3	0	
5F-MMB-PICA	0	0	0	0	0	0	0	1	0	0	0	
ADB-4en-PFUPPYCA	0	0	0	0	0	0	0	0	0	1	0	
ADB-4en-PINACA	0	0	0	0	0	0	0	0	0	1	0	
ADB-BUTINACA	0	0	0	0	0	0	0	0	0	33	1	
ADB-HEXINACA	0	0	0	0	0	0	0	0	0	3	0	
APP-BINACA	0	0	0	0	0	0	1	0	0	0	0	
BB-22	0	0	0	1	0	0	0	0	0	0	0	
MDMB-4en-PINACA	0	0	0	0	0	0	0	11	41	23	7	
MMB-CHMICA	0	0	0	0	1	1	0	0	0	0	0	
Other synthetic cannabinoids												
ADB-INACA^a^	0	0	0	0	0	0	0	0	0	0	1	
CHPIATA^b^	0	0	0	0	0	0	0	0	0	0	2	
Unspecified SCRA	0	0	0	2	1	9	8	0	0	1	0	

^a^ A “no-tail” SCRA, precursor so is not covered by generic legislations in China or the UK., Psychoactivity not yet proven so is not subject to the PSA.

^b^ Has an acetamide linkage so is not covered by the 2021 Chinese generic SCRA, control.

### Opioids

**FIGURE 1:**
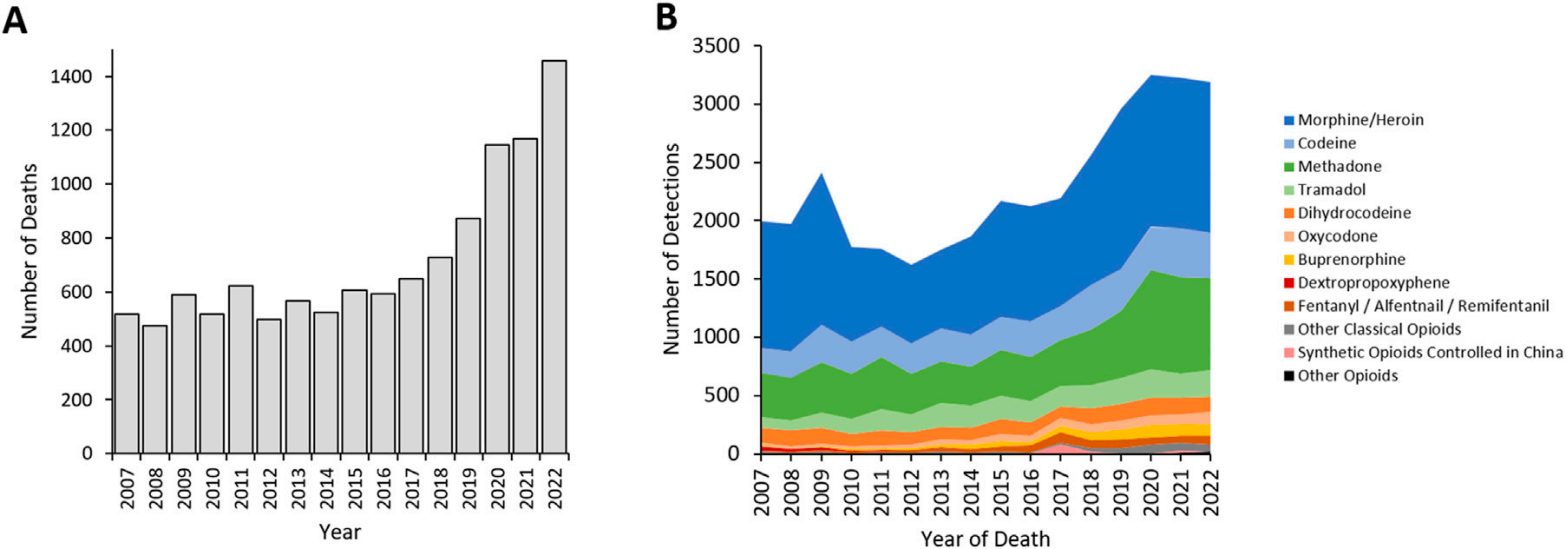
**(A)** Total opioid deaths and **(B)** Detections 2007–2022 reported to the NPSUM. Note: more than one opioid was detected in many cases so the total number of detections in a given year will exceed the total number of deaths.

Deaths with detections of opioids rose in the latter 5 years of the study (2018-2022) with an average of 560 deaths per year over 2007–2017, rising to 1,458 deaths in 2022 (Figure 1A). Classical opioids accounted for the majority of detections in these deaths (Figure 1B; Table 1).

**FIGURE 2:**
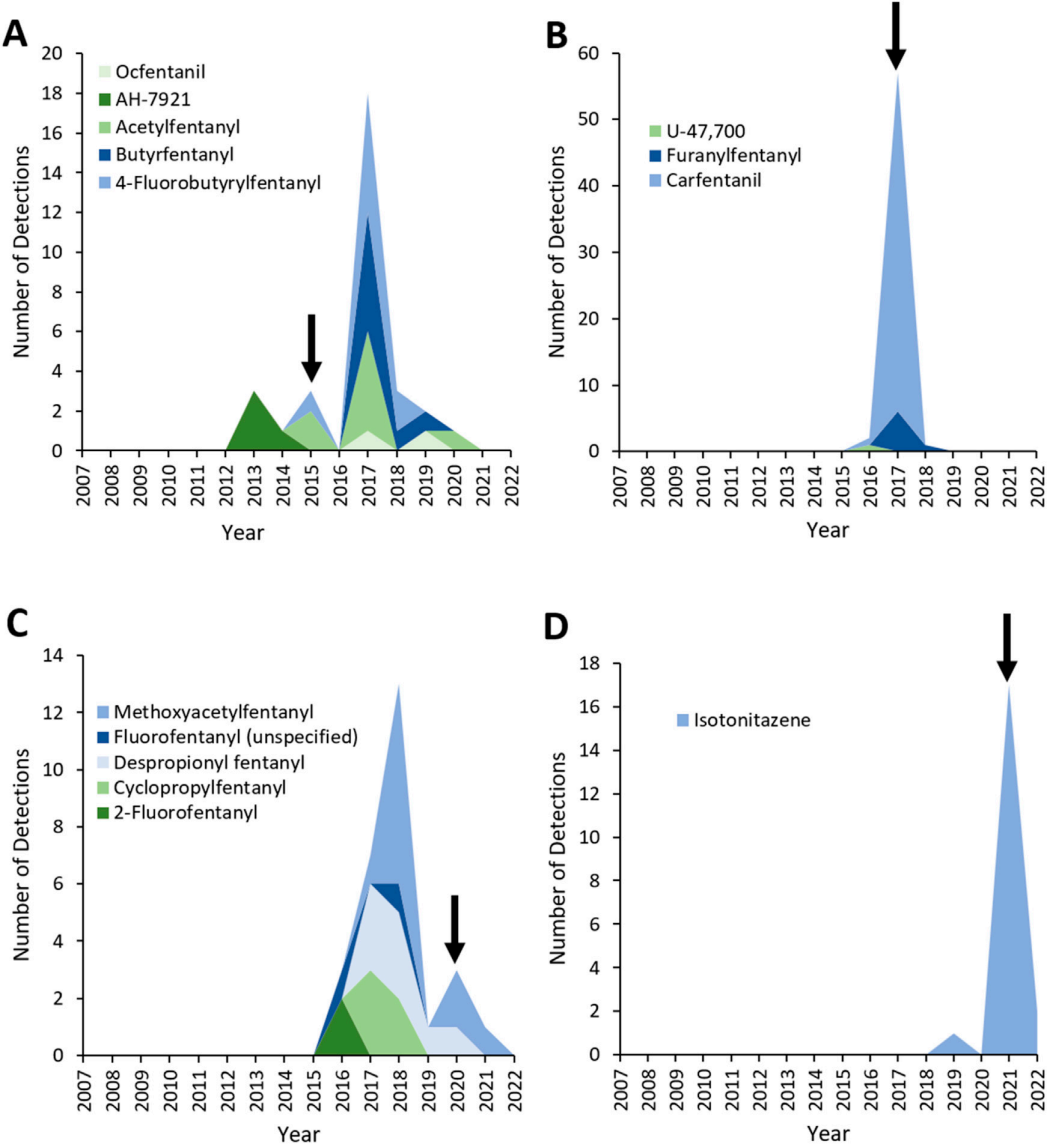
Detections of synthetic opioids controlled in China in **(A)** 2015, **(B)** 2017, **(C)** 2019, **(D)** 2021. Arrows indicate the year where the corresponding drug controls were introduced.

There were no detections of NPS opioids before 2013 (Figures 1B, 2; Table 1). During the subsequent 10-year period, 2013 to 2022, within a total of 24,662 detections of opioids, 254 (1.0%) referred to NPS opioids. NPS opioid detections peaked in 2017 when there were 82 (3.8% of that year’s total detections), primarily driven by a cluster of detections of carfentanil (51 detections, 2.4% of the total).

Five NPS opioids were controlled in China in 2015, four due to specific controls enacted by the Council of China (4-fluorobutyrylfentanyl, acetylfentanyl, butyrfentanyl and ocfentanil) and one due to a UN directed control (AH-7921; Figure 2A; Table 1). Whilst detections of AH-7921 ceased following its ban, the other four NPS opioids controlled in China in 2015 persisted on the UK drugs market, with a peak of 18 detections in deaths occurring in 2017 before disappearing altogether in 2021. These four of NPS opioids had already been controlled for several years in the UK under the MDA (Table 1).

Three further waves of controls on NPS opioids were introduced in China in 2017, 2019, and 2021, with detections of the substances controlled reducing following their enaction (Figures 2B–D). All of the NPS opioids controlled by these Chinese legislations had already been controlled in the UK for several years under the MDA (Table 1).

### Stimulants

Note: In this study, the ‘Ecstasy’ drugs, MDA, MDMA and MDEA, are not regarded as NPS as they were internationally controlled in 1985, 1986, and 1990 respectively (United Nations Office on Drugs and Crime, 1961; United Nations Office, 1971; United Nations Office on Drugs and Crime, 1988). During the study period, there was a marked contribution (69 cases) from para-methoxyamphetamine (PMA) detections. This material has been under international control since 1986 (United Nations Office on Drugs and Crime, 1961; United Nations Office, 1971; United Nations Office on Drugs and Crime, 1988), well before the period of interest, and is therefore not regarded here as an NPS. The closely related para-methoxy methamphetamine (PMMA) contributed a small number of reports (n = 11) concurrent with the PMA reports. PMMA has long been a controlled drug in the UK by virtue of the UK’s 1977 generic control on phenethylamine derivatives (UK Government, 1971). However, as it was not brought under international control until 2016 (United Nations Office on Drugs and Crime, 1961; United Nations Office, 1971; United Nations Office on Drugs and Crime, 1988), it is treated here as an NPS.

**FIGURE 3:**
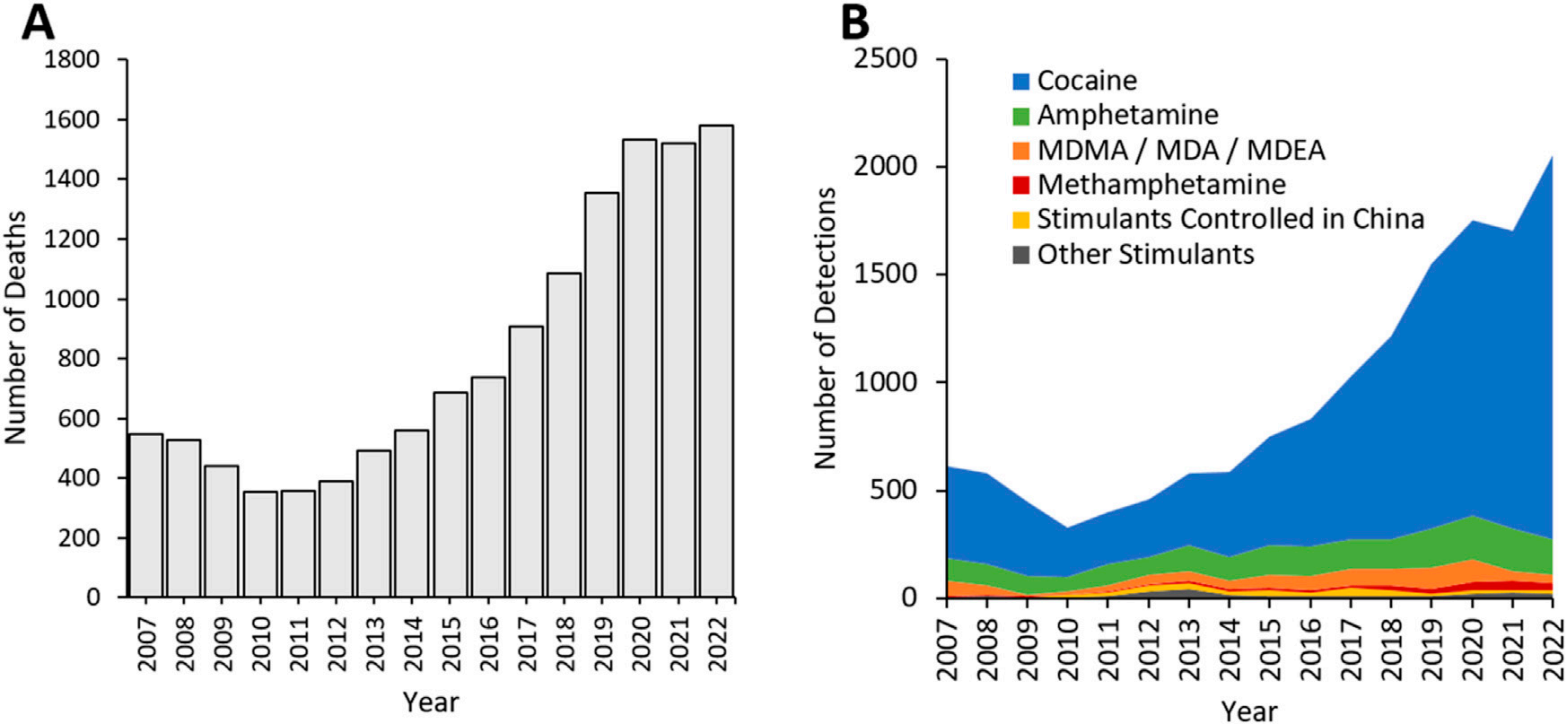
**(A)** Total stimulant deaths and **(B)** Detections 2007–2022 reported to the NPSUM. Note: more than one stimulant was detected in many cases so the total number of detections in a given year will exceed the total number of deaths.

Deaths with detections of stimulants reported to the NPSUM have been steadily rising since 2013, with an average of 436 deaths per year over 2007–2012, rising to 1,581 deaths in 2022 (Figure 3A). Classical stimulants–and then mainly cocaine–accounted for the majority of detections in these deaths (Figure 3B; Table 2): in 2014, cocaine represented 59.3% of stimulant detections, which has risen to 86.4% of detections in 2022.

There were a total of 927 NPS stimulant detections within the study period, representing 6.0% of total stimulant detections. These reached a peak of 114 in 2015, but in the subsequent years in the study, 2016 to 2022, the average number of stimulant NPS detections dropped to approximately 40 per year.

**FIGURE 4:**
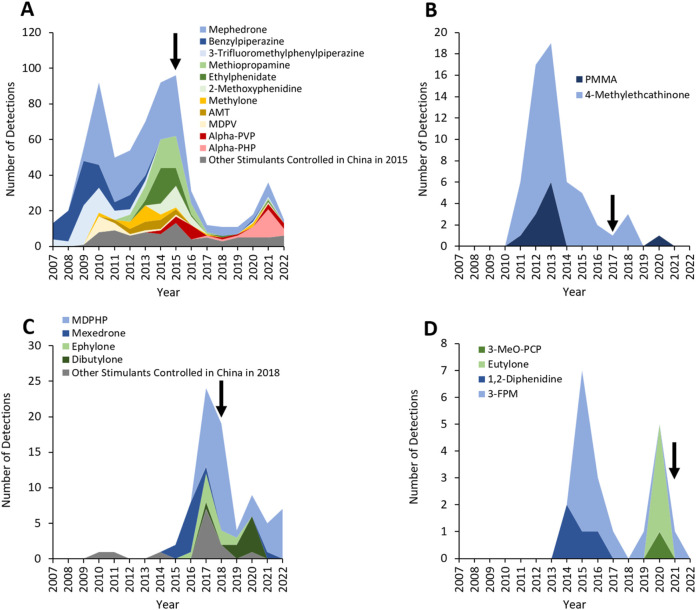
Detections of NPS stimulants controlled in China in **(A)** 2015, **(B)** 2017, **(C)** 2018, **(D)** 2021. Arrows indicate the year where the corresponding drug controls were introduced.

In 2015, the Council of China controlled 116 NPS, 30 of which were stimulants detected in deaths reported to the NPSUM. Whilst 22 of these 30 substances were already controlled in the UK under the MDA (Table 2), it was not until this Chinese legislation was introduced in October 2015 that a considerable drop in detections of these 30 substances was observed (Figure 4A) – however, this drop does also somewhat coincide with the introduction of the PSA in the UK in May 2016. The only exception to this was in detections of alpha-PHP which briefly resurged in 2021 (n = 16).

Three further waves of controls on NPS stimulants were introduced in China in 2017, 2018, and 2021. Whilst detections of these NPS stimulants decreased following introduction of these controls (Figures 4B–D), in some instances decreases were apparent prior to their implementation (e.g., 4-methylethcathinone was controlled in 2016 but detections markedly decreased from 2013; 3-FPM was controlled in 2021 but detection markedly decreased from 2015). All of the NPS stimulants controlled by these Chinese legislations had already been controlled in the UK either under the MDA or PSA (Table 2).

### Cannabinoids

**FIGURE 5:**
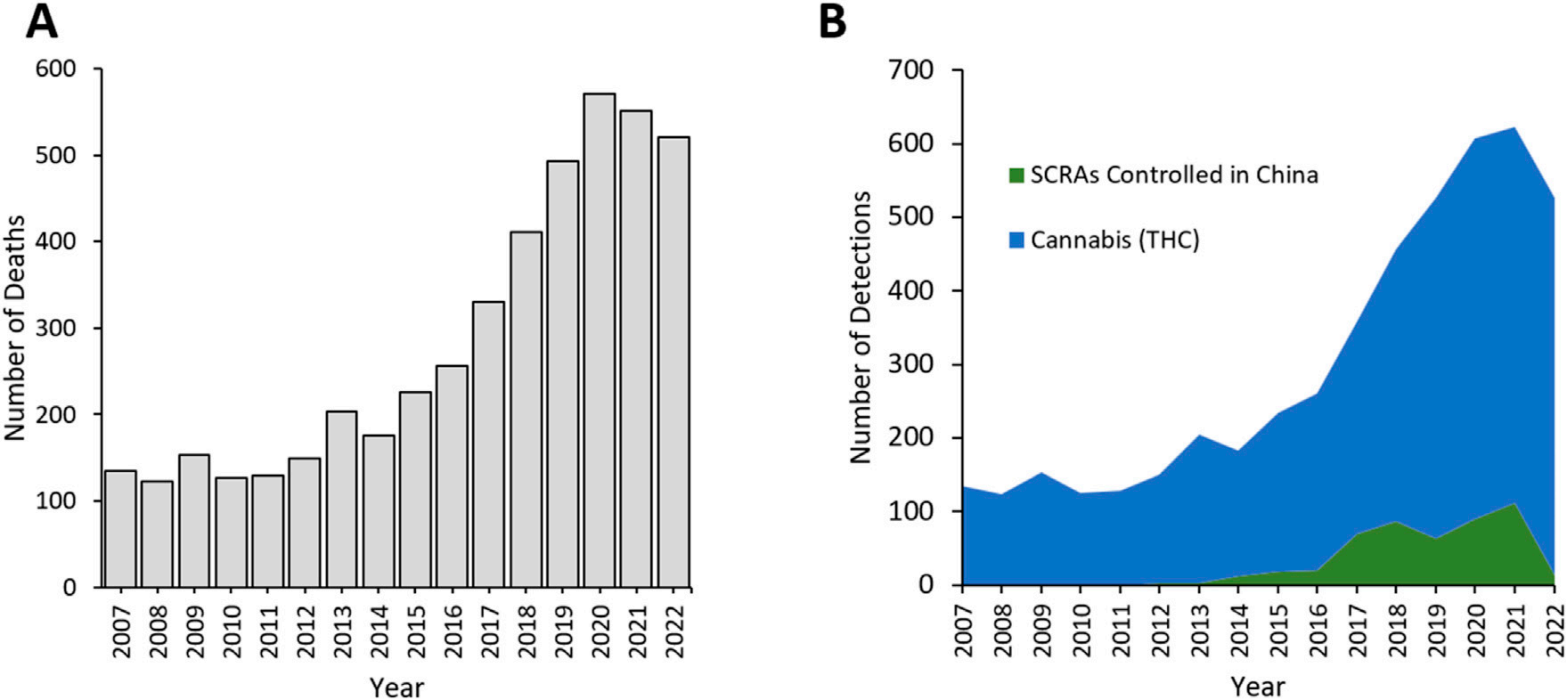
**(A)** Total cannabinoid deaths and **(B)** Detections 2007–2022 reported to the NPSUM. Note: more than one cannabinoid was detected in many cases so the total number of detections in a given year will exceed the total number of deaths.

Deaths with detections of cannabinoids reported to the NPSUM have been steadily rising since 2015, with an average of 149 deaths per year over 2007–2014, rising to 521 in 2022 (although peaking in 2020 at 571 deaths; Figure 5A). Once again, the ‘classical’ cannabinoid detections predominated–in this instance THC as the major psychoactive constituent of cannabis (Figure 5B). The NPS cannabinoids detected are all compounds that fall within the synthetic cannabinoid receptor agonist (SCRA) drug class.

**FIGURE 6:**
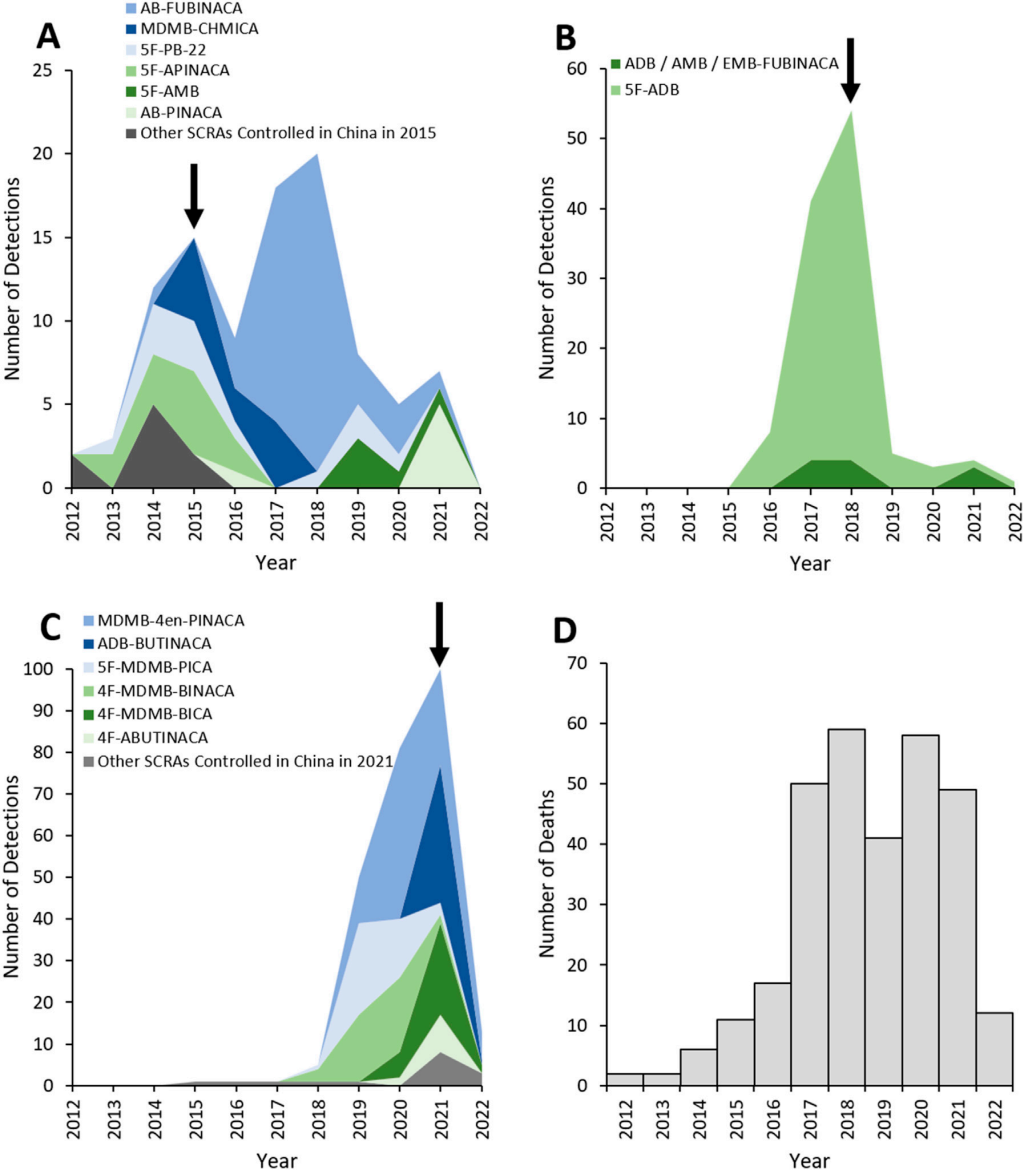
Detections of SCRAs controlled in China in **(A)** 2015, **(B)** in 2018; **(C)** 2021. Arrows indicate the year where the corresponding drug controls were introduced. **(D)** Total number of deaths due to SCRAs reported to the NPSUM.

There were no detections of SCRAs before 2012 (Figures 6A–D; Table 3). A total of 488 detections of 30 different SCRAs were reported to the NPSUM over the study period, first peaking in 2018 with 87 detections and then again in 2021 with 112 detections, before drastically falling in 2022 to 14 detections (from 12 deaths; Figure 6D; Table 3). Six SCRAs accounted for 63.5% of these detections: 5F-MDMB-PINACA (also known as 5F-ADB) 21.5%, MDMB-4en-PINACA 16.8%, AB-FUBINACA 9.0%, 5F-MDMB-PICA 8.2%, 4F-MDMB-BINACA 8.0% and ADB-BUTINACA 7.0%.

The first SCRAs were controlled in China in 2015, following which detections of those controlled dropped, except for AB-FUBINACA (Figure 6A; Table 3). Of the 11 SCRAs which were controlled in China in 2015 and which were detected in deaths, only two were already controlled in the UK under the MDA (Table 3).

Two further waves of controls on SCRAs were introduced in China in 2018 and 2021, with the 2021 legislation detailing a generic control on compounds featuring the skeleton SCRA structure. Detections of 5F-ADB and ADB/AMB/EMB-FUBINACA decreased following their control in China in 2018 (Figure 6B), as did detections of other SCRAs that had subsequently emerged following introduction of the 2021 generic SCRA control (Figure 6C). All of the SCRAs controlled by these latter Chinese legislations had already been controlled in the UK under the PSA (and many subsequently under the MDA; Table 3).

## Discussion

In this study we have provided the first systematic evaluation of the relative impacts of UK, Chinese, and UN legislative controls on the availability of NPS in England, Wales and Northern Ireland, evidenced by their post-mortem detections in deaths following psychoactive drug use. By linking toxicological evidence to the introduction of drug control legislations across these jurisdictions, we were able to infer which regulatory controls were most effective in influencing the most negative effect (i.e., death) of NPS available on the UK drug market.

### ‘Classical’ substances remain the dominant drivers of drug-related mortality

A consistent finding across the three drug classes studied–opioids, stimulants and cannabinoids–is that classical substances were detected in the vast majority of deaths, eclipsing those with involvement of NPS. This illustrates that whilst NPS attract significant attention due to their novelty, unpredictable harms, and the challenges that they pose for regulators (Deen et al., 2021; Home Office, 2011; Home Office, 2018; Rinaldi et al., 2020; Nutt, 2020), drug-related mortality continues to be driven by established, widely available substances (National Statistics, 2024). Legislative controls on NPS have therefore not reduced overall demand for psychoactive substances, but rather reshaped the composition of the illicit drug market with a renewed consumption of classical substances.

### UK vs. Chinese legislative impacts

Whilst the majority of UK legislative controls on NPS were enacted earlier than either the Chinese or UN equivalents, they appear to have had limited impact on the appearance of NPS in deaths. Instead, reductions in NPS detections correlated more with the introduction of Chinese controls. This is consistent with the central role that Chinese manufacturing and export networks are thought to hold in supplying the global NPS market (Zhao, 2022; Zhao, 2020; Bao et al., 2019; Deventer Marie et al., 2025; Seddon, 2014; Gomes and Rudkowsky, 2025).

For NPS opioids, UK controls appear to have been largely ineffective, whereas the controls of these substances in China in 2017, 2019 and 2021 correspond with sharp reductions in their detection. An exception to this was the 2015 Chinese opioid controls, which were followed by a persistence of four non-pharmaceutical fentanyl analogues in deaths up until 2021. This anomaly could be due to a number of reasons, including that the 2015 controls represented one of the earliest and largest waves of Chinese NPS legislation (Ministry of Public Security of the People’s Republic of China, 2015) which may not have been enforced as comprehensively as subsequently introduced controls, that these fentanyl analogues may have been produced outside of China, or stockpiled materials within the UK. These explanations remain speculative however, in the absence of direct evidence. With regards to NPS stimulants, a similar pattern is evident: whilst the UK had already controlled the majority of cathinones under the MDA, there was only a marked drop in detections after China controlled many stimulant NPS in 2015. The clearest evidence of Chinese legislative impact was observed with cannabinoid NPS as the 2021 generic ban on SCRAs virtually eliminated the presence of these compounds in UK deaths within the year, reducing detections from over 100 to just 14 by 2022. In contrast, the earlier UK controls on SCRAs via the MDA and PSA had limited impact on the appearance of successive generations of SCRAs.

Taken together, these findings suggest that controlling NPS at their point of origin is more effective than restricting access and availability at the point of demand. This raises a pertinent and pressing question for future UK drug policy on emergent NPS: how can the UK move beyond a reactive position–dependent upon which NPS emerge from producer countries–to more proactively reduce the harms inflicted by the substances which ultimately reach its market?

### Unanticipated consequences of NPS controls on market shifts

Prior to the implementation of the PSA, deaths with detections of cocaine were comparatively lower, suggesting that some NPS stimulants may be associated with a lower incidence of lethal toxicity than cocaine. Indeed, a number of pre-clinical studies have found several stimulant NPS–and particularly cathinone derivatives–to exert weaker dopaminergic activities when compared with cocaine (Opacka-Juffry et al., 2014; Kelly, 2011; López-Arnau et al., 2012; Simmler et al., 2013). Following the ban of stimulant NPS under the PSA, stimulant users appear to have returned to classical substances–and in particular cocaine–which was followed by a sharp rise in cocaine detections in deaths. A similar pattern was observed in Germany following the introduction of the Neue-psychoaktive-Stoffe-Gesetz (NpSG) in 2016 –legislation which generically controlled NPS rather than individual substances similar to the PSA–as there was a marked decline in the detection of NPS (Zellner et al., 2024; Sommer et al., 2022; EUDA, 2025) suggestive of a reduction in their domestic availability. However, subsequent studies have indicated that many German NPS users returned to using classical substances (EUDA, 2025; Kühnl et al., 2022), thus mirroring what was observed in the UK. This trend is illustrative of a recurring theme across drug markets which has been termed the ‘potency paradox’ (Thornton, 1998; Biedermann, 2017) in which restrictions on one substance/class of substances may be leading unintentionally to increased harms by steering users towards more potent alternatives. Indeed, the control of ketamine in the UK as a Class C substance in 2006 (and subsequently as a Class B substance in 2014) (UK Government, 1971) was followed by the appearance of methoxetamine and diphenidines (Chiappini et al., 2015; Corkery et al., 2025), which were marketed as legal replacements to ketamine, but possess greater potency and longer duration of action (Horsley et al., 2016; Luethi et al., 2018; Wallach et al., 2016) leading them to be associated with more severe toxicological outcomes (ACMD, 2012). A related phenomenon is now playing out within the opioid drug class: although in this analysis NPS opioids represented only a fraction of detections, the subsequent widespread arrival of nitazenes in the UK in 2023 (Holland et al., 2024) after the end of the study period (note: the UK and China both implemented generic controls on nitazenes in 2025 (UNODC, 2025; Ministry of Public Security of the People’s Republic of China, 2025), however, it is not yet possible to assess the impact of these controls via analysis of drug deaths) has been linked to restrictions on less potent within-class alternatives (e.g., fentanyl analogues) and reduced heroin availability from Afghanistan (Holland et al., 2024). Together, these findings highlight a central drug policy dilemma: whilst prohibition can reduce availability of NPS, it may also be a driver towards other more harmful substances, illustrating the need for complementary harm reduction strategies to be delivered alongside the implementation of legislative controls.

### Strengths and limitations

This study has both strengths and limitations. A strength of this study is that it used routinely collated post-mortem toxicology data from the NPSUM, covering over 85% of coronial jurisdictions in England, Wales and Northern Ireland. This has provided a unique, mortality-based perspective of the NPS market, which is arguably one of the most clinically-relevant outcomes. However, the voluntary nature of reporting means that not all deaths, and therefore NPS detections, will have been captured. Furthermore, NPS toxicology screens are not requested in every case subject to coronial investigation, differ in terms of the screening library between laboratory operators, and will have happened at a particularly low incidence in the earlier years of the study when awareness of NPS was not as widespread. This may therefore have significantly impacted upon when NPS were first detected, as if not screened for they will not have been reported. However, as our analyses primarily focus on when NPS ceased to be detected in relation to legislative changes - by definition, such substances must have been previously included in analytical screens. The linking of mortality data to legislative implementations across multiple jurisdictions has allowed for stronger inference on the likely impact of different regulatory implementations. However, causality cannot be inferred with certainty: whilst temporal associations between NPS controls in China and reduced detections are strong, other market forces will have also contributed (e.g., law enforcement strategy, changing user preferences, price shifts). In this regard, it is important to note that NPS legislation introduced in India was not included as an additional variable in this analysis, despite India’s growing role as a producer of NPS (EUDA, 2025). This is because the legislations directing control of specific NPS in India were introduced either at the same time or after the same compounds had been directed for control under the UN Conventions, so that India, as a signatory of the Conventions, was obliged to enact controls (Narcotics Control Bureau, 2017; Narcotics Control Bureau, 2018; Narcotics Control Bureau, 2015; Narcotics Control Bureau, 2019; Narcotics Control Bureau, 2021; N arcotics Control Bureau, 2021). Finally, as this analysis focused only on deaths, it has not captured trends in non-fatal NPS use. However, a recent UK study on acute recreational drug toxicity presentations in London which compared two time-points (2016/17 vs. 2019/20) had complementary findings to the present study as they observed a reduction in cathinone detections but no significant change in SCRA detections between these two timepoints (Wolfe et al., 2025). This suggests that trends in non-fatal NPS toxicity presentations broadly reflect those observed in the mortality data.

## Conclusion

The findings of this study indicate that the most effective way to reduce NPS availability in the UK is via legislation in producer countries, as evidenced by substantial declines in their detections in deaths following their control in China. UK interventions–despite enabling national law-enforcement activity and often being enacted earlier–had limited impact, highlighting the restricted influence of consumer country legislation in a globalised market. This reliance on international controls places the UK in a vulnerable position, as its domestic drug landscape is being shaped largely by the pace and scope of independent international legislations. To achieve and maximise effectiveness, UK drug policy needs to integrate harm reduction measures alongside the introduction of legislative controls, whilst also encouraging international efforts to bring in global control of problem materials. Without this combined approach, the cycle of NPS emergence, prohibition, and displacement will continue to undermine public health.

## Data Availability

Original data can be obtained from the corresponding author upon reasonable request. Requests to access these datasets should be directed to caroline.copeland@kcl.ac.uk.
